# Simple sequence repeat markers useful for sorghum downy mildew (*Peronosclerospora sorghi*) and related species

**DOI:** 10.1186/1471-2156-9-77

**Published:** 2008-11-29

**Authors:** Ramasamy Perumal, Padmavathi Nimmakayala, Saradha R Erattaimuthu, Eun-Gyu No, Umesh K Reddy, Louis K Prom, Gary N Odvody, Douglas G Luster, Clint W Magill

**Affiliations:** 1 Department of Plant Pathology and Microbiology, Texas A&M University, College Station, Texas – 77843-2132, USA; 2 Department of Plant Pathology and Microbiology, Texas Agrilife Sciences, Corpus Christi, Texas – 78406-1412, USA; 3 Institute for Plant Genomics & Biotechnology, Texas A&M University, College Station, Texas – 77843-2123, USA; 4 West Virginia State University, Institute, Department of Biology and Gus R. Douglas Institute, WV – 25112, USA; 5 USDA-ARS, Southern Plains Agricultural Research Center, College Station, Texas – 77845, USA; 6 USDA/ARS, Foreign Disease-Weed Science Research Unit, Ft. Detrick, Maryland – 21702, USA

## Abstract

**Background:**

A recent outbreak of sorghum downy mildew in Texas has led to the discovery of both metalaxyl resistance and a new pathotype in the causal organism, *Peronosclerospora sorghi*. These observations and the difficulty in resolving among phylogenetically related downy mildew pathogens dramatically point out the need for simply scored markers in order to differentiate among isolates and species, and to study the population structure within these obligate oomycetes. Here we present the initial results from the use of a biotin capture method to discover, clone and develop PCR primers that permit the use of simple sequence repeats (microsatellites) to detect differences at the DNA level.

**Results:**

Among the 55 primers pairs designed from clones from pathotype 3 of *P. sorghi*, 36 flanked microsatellite loci containing simple repeats, including 28 (55%) with dinucleotide repeats and 6 (11%) with trinucleotide repeats. A total of 22 microsatellites with CA/AC or GT/TG repeats were the most abundant (40%) and GA/AG or CT/TC types contribute 15% in our collection. When used to amplify DNA from 19 isolates from *P. sorghi*, as well as from 5 related species that cause downy mildew on other hosts, the number of different bands detected for each SSR primer pair using a LI-COR- DNA Analyzer ranged from two to eight. Successful cross-amplification for 12 primer pairs studied in detail using DNA from downy mildews that attack maize (*P. maydis & P. philippinensis*), sugar cane (*P. sacchari*), pearl millet (*Sclerospora graminicola*) and rose (*Peronospora sparsa*) indicate that the flanking regions are conserved in all these species. A total of 15 SSR amplicons unique to *P. philippinensis *(one of the potential threats to US maize production) were detected, and these have potential for development of diagnostic tests. A total of 260 alleles were obtained using 54 microsatellites primer combinations, with an average of 4.8 polymorphic markers per SSR across 34 *Peronosclerospora, Peronospora and Sclerospora *spp isolates studied. Cluster analysis by UPGMA as well as principal coordinate analysis (PCA) grouped the 34 isolates into three distinct groups (all 19 isolates of *Peronosclerospora sorghi *in cluster I, five isolates of *P. maydis *and three isolates of *P. sacchari *in cluster II and five isolates of *Sclerospora graminicola *in cluster III).

**Conclusion:**

To our knowledge, this is the first attempt to extensively develop SSR markers from *Peronosclerospora *genomic DNA. The newly developed SSR markers can be readily used to distinguish isolates within several species of the oomycetes that cause downy mildew diseases. Also, microsatellite fragments likely include retrotransposon regions of DNA and these sequences can serve as useful genetic markers for strain identification, due to their degree of variability and their widespread occurrence among sorghum, maize, sugarcane, pearl millet and rose downy mildew isolates.

## Background

Sorghum (*Sorghum bicolor*) is the world's 5^th ^most important grain crop [1], and with a genome much smaller than maize, is a model for tropical grasses of worldwide importance. It is a major source of food, feed, fiber, fuel, and chemical feed stocks across a range of environments and production systems. However, diseases and insects, in addition to abiotic stresses, are major impediments to sorghum production. Among the diseases, sorghum downy mildew (SDM), caused by *Peronosclerospora sorghi*, [2] is a disease of sorghum and maize (*Zea mays*) [3]. SDM can cause severe epidemics, resulting in heavy yield loss. In the past *P. sorghi *has been subdivided into 'sorghum/maize' and 'maize' infecting strains. As more information has become available, the *Peronoscleosporas *have been designated as separate species, with names that indicate the preferred host (*P. maydis *on maize and *P. sacchari *for sugarcane). Although *P. sorghi *can produce symptoms on both maize and sorghum, it typically does not complete sexual reproduction on maize, so no oospores are formed. Sorghum and maize downy mildew (*P. sorghi*) reported in 44 countries and pearl millet downy mildew (*Sclerospora graminicola*) reported in 51 countries around the globe are found in most tropical and subtropical regions [4,5]. These oomycetes are both seed and soil-borne pathogens, thus rendering crop rotation less effective in controlling the disease. A sudden re-emergence and disease outbreak of sorghum downy mildew resulting in significant yield loss was noted in Wharton and Jackson counties of Texas during the spring of 2001 and again in 2002 [6]. *P. sorghi *isolates from the outbreak were found to be resistant to metalaxyl [methyl N- (2, 6-dimethylphenyl)-N-(methoxyacetyl)-DL-alaninate] fungicide, which had been used as an effective seed treatments for many years. Subsequently, isolates collected from previously resistant hosts revealed the evolution of a new pathogenic race of *P. sorghi *and demonstrated a need for constant monitoring of the pathogen populations.

Philippine downy mildew of maize, caused by *Peronosclerospora philippinensis *(Weston) C.G. Shaw, has not been reported in the western hemisphere, but it is a serious problem in the Philippines where disease losses in some fields have been 40–60% [7]. Although maize varieties resistant to *P. philippinensis *have been developed in Asia, maize hybrids currently grown in the United States are highly susceptible to *P. philippinensis *and American breeding lines that are highly resistant to *P. sorghi *in the United States are generally susceptible to *P. philippinensis *(M.R. Bonde, personal communications). Besides maize and sorghum [8], *P. philippinensis *has been reported to infect *Avena sativa *L., *Saccharum officinarum *L., *Sorghum halepense *(L.) Pers, *S. propinquum *(Kunth) Hitchc. [9] and *Saccharum spontaneum *L. [10]. Consequently, *P. philippinensis *is the subject of quarantine in many countries and was listed by USDA APHIS under CFR 7 part 331 as a "select agent" in the US, meaning it is considered to be a major threat to agricultural security. Because of the threat posed by *P. philippinensis*, a DNA sample available from the USDA, ARS Foreign Disease-Weed Science Research Unit at Ft. Detrick, MD was included in this study. The information gained, besides having epidemiologic significance for maize and sorghum production, also could help resolve confusion over the taxonomy of *Peronosclerospora *spp.

The population structure of *P. sorghi *has been previously addressed with physical characters, such as spore size or pathotype, which require comparative disease responses on a series of host differential cultivars [11,12]. Conventional methodologies for the detection of different pathotypes of downy mildews of cereals have not been satisfactory particularly for the identification of organisms at extremely low propagule numbers and are cumbersome for use with obligate parasites (such as plant-pathogenic rusts and mildews), which cannot be cultured. Hence, in tropical climates, symptoms of *Peronosclerospora *can be confused with those from related downy mildew pathogens, including *P. maydis, P. sacchari *and *P. philippinesis*. Yao et al. [13] showed that clones of *P. sorghi *DNA cross-hybridized well on Southern blots with DNA from the other *Peronosclerospora *species. While few differences were detected among the RFLP patterns within *P sorghi*, different patterns were seen for the different species. Similarly, ITS-2, an internal transcribed spacer of ribosomal genes was different in size [14]. While potentially useful in species identification, more easily scored and highly variable markers from locations throughout the genome are needed for differentiating among closely related pathotypes, and to tag genes that contribute to host specificity, race specificity, and virulence. Although polymorphisms were detected in SDM pathotypes using RAPD-PCR, many of the short (10 mer) primers resulted in monomorphic banding patterns. A larger problem involved inconsistently amplified bands that varied greatly in intensity, even in duplicate experiments [15]. As a consequence, a more consistent procedure was sought in the present investigation for the development of microsatellite markers.

Simple sequence repeat (SSR), or microsatellites, are hypervariable and dispersed in the form of long arrays of short tandem repeat units throughout the genome [16,17]. These SSRs provide codominant Mendelian markers, much more powerful than dominant markers and can be used to determine population genetic structure, kinship, reproductive mode, and genetic isolation [18,19]. When compared with several other marker types, SSR markers were superior for genetic characterization in *Aspergillus fumigatus*, and in *Saccharomyces cerevisiae *[20,21]. Microsatellite markers have been developed for several fungi including *Magnaporthe grisea *[22,23], and another oomycete, *Phytophthora infestans *[24] among others. However, based on sequence searches using genome data *P. infestans *and two other plant pathogenic *Phytophthoras*, potential SSRs have been reported to be rare [25].

In the present study, many new microsatellite sequences comprised of dinucleotide and trinucleotide repeat structures were retrieved from genomic DNA of Sorghum Downy Mildew (*Peronosclerospora sorghi*) pathotype 3 using an optimized and highly simplified biotin capture protocol. On the basis of sequence analysis of these captured fragments, appropriate primers were designed for amplification of these microsatellite loci and the developed microsatellites for *P. sorghi *were tested for the ability to differentiate pathogenically diverse isolates of *P. sorghi *and for their utility for cross-species amplification in *P. philippinensis*, *P. maydis *(maize), *P. sacchari *(sugar cane), *Sclerospora graminicola *(pearl millet) and *Peronospora sparsa *(rose) downy mildew. Clustering analysis was performed to assess the genetic diversity and close relatedness among 34 isolates including the different species of downy mildews.

## Methods

### Pathogen samples and DNA isolation

A total of 19 isolates of *Peronosclerospora sorghi *including P1 (metalaxyl susceptible), P3 and P6 (both metalaxyl resistant and susceptible) pathotypes collected in different years from different parts of Texas where the disease outburst occurred were included in this study. In addition, DNA samples from *P. philippinensis *(one), *P. maydis *(five), *P. sacchari *(three) were obtained from Dr. Douglas G. Luster, USDA/ARS, Foreign Disease-Weed Science Research Unit, Ft. Detrick, Maryland, USA and DNA samples of *Sclerospora graminicola *(five) and *Peronosopora sparsa *(one) available in Dr. Magill's lab, Dept. of Plant Pathology & Microbiology, Texas A&M University, College Station, Texas, USA, were also included in the present study (Table 1). For DNA isolation, conidia were collected from infected leaves as follows: leaves were washed with cold sterile water and placed abaxial side down on a screen in a petri plate, covered with moist paper towels and allowed to sporulate in the dark overnight at 23°C [26]. Spores collected in sterile water were used for DNA extraction. After removing excess water, conidial suspensions were washed a few times with 70% ethanol and frozen for lyophilization. The dried sample was powdered in liquid nitrogen using a mortar and pestle. DNA was extracted using a MasterPure Yeast DNA Purification kit (EPICENTRE Biotechnologies, Madison, WI) and the DNA was diluted to working concentration of 2.5 ng/μL by adding 1× TE (10 m M Tris-HCl, 1 mM EDTA, pH 8.0).

**Table 1:** Downy mildew isolates of *Peronosclerospora*, *Sclerospora *and *Peronospora *spp analyzed in this study

***Peronosclerospora sorghi *– Sorghum downy mildew isolates**				
Iso. No	Pathotype/DNA	Metalaxyl reaction	Host	Location & year

1	P1	Sensitive	Tx7978-sorghum	Green house, TAMU, TX

2	P3	Sensitive	Tx430-sorghum	Green house, TAMU, TX

3	P3	Sensitive	Whart-A-sorghum	Wharton County, TX – 2001

4	P3	resistant	CR 360A – sorghum	Wharton County, TX – 2002

5	P3	resistant	CR 459A – sorghum	Wharton County, TX – 2002

6	P3	resistant	Fucik B – sorghum	Wharton County, TX – 2002

7	P3	resistant	Merta A – sorghum	Wharton County, TX – 2002

8	P3	resistant	Wesla A – sorghum	Weslaco, TX – 2002

9	P3 or P6	resistant	Grain sorghum	Wharton County, TX – 2004

10	P6	resistant	Johnson grass	Jackson county TX – 2007

12	P6	resistant	Grain sorghum	Wharton County, TX – 2007

13	P6	resistant	Grain sorghum	Wharton County, TX – 2006

14	P3	resistant	Grain sorghum	Wharton County, TX – 2006

15	P3 or P6	resistant	Grain sorghum	Wharton County, TX – 2006

16	P1	Unknown	Johnson grass	Nueces county, TX – 2007

17	P6	resistant	Johnson grass	Wharton county, TX – 2007

18	P6	resistant	Grain sorghum	Wharton county, TX – 2006

19	P3 or P6	resistant	Grain sorghum	Jackson county, TX – 2006

21	P1	Sensitive	Grain sorghum	Nueces county, TX – 2007

***Peronosclerospora maydis *– Maize downy mildew isolates^§^**				

22	DNA # 5	-	Corn	Suwan Farm, Thailand-1975

23	DNA # 6	-	Corn	Malang, Indonesia – 1987

24	DNA #. 7	-	Corn	West Java, Indonesia – 1987

25	DNA #. 8	-	Corn	Suwan Farm, Thailand-1985

26	DNA # 9	-	Corn	Suwan Farm, Thailand-1985

***Peronosclerospora philippinensis*isolate^§^**				

27	DNA #. 1	-	Corn	LosBanos, Phillippines, 1979

***Peronosclerospora sacchari *– Sugarcane downy mildew isolates^§^**				

28	DNA # 2	Race I – Sugar cane		China – 1975

29	DNA # 3	Sugar cane		Taiwan – 1975

30	DNA # 4	Isolate 77B-SCI – Sugar cane		Taiwan – 1977

***Peronospora sparsa *– Rose downy mildew isolate**				

31	DNA#1	Infected Rose plant		Dis. Diagno. Lab, TAMU, TX

*Schlerospora graminicola *– Pearl millet downy mildew isolates*				

32	DNA #6	cultivar GK1004 – Pearl millet		Kennola, A'bad Dt, India

34	DNA #37	cultivar MLPH 104 – Pearl millet		Vadagaon, A'nagar Dt., India

36	DNA #42	Local cultivar – Pearl millet		Kawdiyal, Bidar Dt., India

37	DNA #16	cultivar Proagro – Pearl millet		Hatnur, A'bad Dt., India

38	DNA #20	Cultivar Vijay-4-Pearl millet		Parola, Jalgoan Dt., India

* Sporangial isolates 32 through 38 collected from different locations in major pearl millet growing areas in Maharastra, India, Aug. 1999 and were received through ICRISAT (International Crop Research Institute for Semi-Arid Tropics), Patancheru – Hyderabad 502 324, India.

^§ ^DNA samples received from USDA/ARS, Foreign Disease-Weed Science Research Unit, Ft. Detrick, MD 21702.

### Microsatellite Isolation

A microsatellite-enriched library was prepared by a simplified protocol based on the biotinylated-oligonucleotide capture methods of [27]and [28]. Further, several steps in the biotin capture protocol were modified as detailed by Reddy et al. [29] to optimize the frequency of microsatellite repeats among captured genomic DNA fragments. In our protocol, no size fractionation steps or radioactive hybridizations were employed. A 500 ng sample of genomic DNA from SDM pathotype 3 was digested for 3 h in a single reaction mixture containing restriction endonucleases *Hae*III, *Rsa*I, and *Dra*I (20 units of each), as well as 50 ng of RNaseA. This digestion resulted in a diverse population of blunt-ended restriction fragments with an average size of ~550 bp. Digested DNAs were purified using a QIA-quick PCR purification column (Qiagen, Valencia, CA), eluted with 50 μl of 5 mM Tris-pH 8.0, then dried completely under vacuum. The double-stranded adaptor molecule AP11/12 was prepared by mixing equal molar amounts of oligonucleotides AP11 (5'CTCTTGCTTAGATCTGGACTA3') and AP12 (5'pTAGTCCAGATCTAAGCA-AGAGCACA3', where p = 5' phosphate), heating to 94°C, then cooling to 25°C over a period of 5 h. Digested genomic DNA fragments were resuspended in a 30-μL ligation reaction containing 100 ng of AP11/12 double-stranded adaptor and 30 Weiss units of T4 DNA ligase. Ligation was carried out at 14°C for 16 h. Preamplification of adaptor-ligated products was performed using 2 μL of the ligation reaction as a template for 10 cycles of PCR in a 50-μL reaction volume using the single primer AP11. An annealing temperature of 55°C was employed in all PCR reactions.

Approximately 100 ng of the preamplified product was then added to a single reaction mixture containing 6× SSC (0.9 M NaCl, 90 mM sodium citrate, pH 7), 0.1% SDS (sodium dodecyl sulfate), and 200 ng each of biotinylated oligos b(TA)30, b(CA)20, b(GA)20, and b(AGA)15, b(TGA)15, b(ACA)15, (b = 5' biotinylation). After denaturation at 95°C for 5 min, preamplified genomic DNA fragments were annealed in the presence of biotinylated oligonucleotides for 1 h at 60°C, then added to 200 μg of fresh streptavidin-coated paramagnetic beads (Promega, Madison, WI) previously equilibrated with 6× SSC. Beads were incubated at 60°C with gentle agitation for 15 min, then the liquid was removed by separation using a magnetic stand (Stratagene, San Diego, CA). Beads were washed twice in 300 μL of 6× SSC, 0.1% SDS for 15 min at room temperature with gentle agitation. Beads were further washed twice in 300 μL 6× SSC, 0.1% SDS for 15 min at 60°C with gentle agitation. Finally, beads were briefly washed twice with 6× SSC at room temperature. After removing the final wash, captured DNAs were eluted from the beads with the addition of 100 μL of 60°C 0.1 M NaOH. After neutralization with 100 μL of 1 *M *Tris-pH 7.5, captured DNAs were desalted and equilibrated with 10 m*M *Tris-pH 8.0, 1 m*M *EDTA-pH 8.0 (to a final volume of ~50 μL) using a 100-kDa MW cutoff size filtration column (Millipore, Bedford, MA). Five μL of desalted DNA sample were used as a template for 30 cycles of PCR in a 50-μL reaction volume using primer AP11. Six microliters of the resulting PCR reaction (~60 ng) was cloned into the TA-cloning vector pCR4-TOPO through topoisomerase mediated ligation (Invitrogen, San Diego, CA) and transformed into chemically competent *Escherichia coli *TOPO10. Recombinant colonies were identified by positive selection through insertional inactivation of the *ccd*B (control of cell death) open reading frame. Colonies were transferred to 96-well microtiter plates for archival storage.

### Sequence Analysis

Recombinant bacterial colonies were inoculated into 300 μL of LB amp media in a 96-well 0.6-mL-deep plate (Marsh Bioproducts, Rochester, NY). Cultures were agitated at 500 rpm in a HiGro high-density shaking-incubator (GeneMachines, San Carlos, CA) for 16 to 18 h at 37°C. DNA sequencing was performed by RCA (rolling circle amplification) using Templiphi 100 Amplification Kit (GE Healthcare Bio-Sciences Co. Piscataway, NJ). 0.5 μl of the bacteria was added into 5 μl of sample buffer. The sample was denatured at 95°C for 3 minutes, and then cooled to room temperature or 4°C. The denatured sample was combined with 5 μl of reaction buffer and 0.2 μl enzyme mix. DNA was incubated at 30°C for 18 hrs for Templiphi reaction. The amplified DNA was diluted 50 ul of water. 2 ul from the diluted RCA product was sequenced using BigDye terminator cycle sequencing (Perkin-Elmer Applied Biosystems, Foster City, CA) using 10 pmol of M13-forward and M13-reverse primer in a 10-μL reaction. Standard cycle sequencing conditions were employed. Sequencing products were purified by DyeEX 96 kit (Qiagen, Valencia, CA). Electrophoretic separation of sequencing products was performed on an ABI Prism 3130xl Genetic Analyzer (Applied Biosystems, Foster City, CA). Assembly of double-stranded DNA sequence contigs from each clone, and identification of redundancy and overlaps between clones was performed using Sequencher 3.0 (Gene Codes, Ann Arbor, MI). Microsatellite analysis for the identification of sequences with perfect and imperfect repeats and simple and compound repeat motifs were identified using Simple Sequence Repeat Identification Tool (SSRIT) database [30].

### Sequence homology search

The sequences of SDM microsatellites were subjected to homology searches using BLASTN against nucleotide collection (nr/nt) and Expressed Sequence Tags (est_others) at National Center for Biotechnology Information (NCBI; [31]).

Under *Algorithm parameters *the following options were chosen to improve the results.

### General parameters

Max target sequences – 100; Short queries – automatically adjust parameters for short input sequences; Expected threshold – 10 & Word size – 11

### Scoring parameters

Match/Mismatch scores – 2,-3; Gap costs – Existence: 5 Extension: 2

### Filters and Masking

Filter – low complexity regions; species-specific repeats for fungi

Mask – Mask for lookup table only options.

### Design of primers and PCR amplification

Primers were designed to the flanking region of the SSR using PRIMER 3 software[32]. Primers were designed without repetitive DNA and with a base composition of greater than 40% G+C with annealing temperature between 52°C and 58°C to yield amplification product between 100 and 250 bp. Oligonucleotide primers were synthesized by Integrated DNA Technologies, Coralville, IA, USA. Primers selected by these criteria were evaluated further for melting temperature, internal structure, and propensity for primer-dimer formation using publicly accessible Worldwide Web resources (Sigma-Genosys, The Woodlands, TX). PCR amplification was carried out in 10 μL containing 5.5 ng of genomic DNA, 1 μL 10× PCR buffer (Promega), 0.8 μL of 2.5 mM dNTP mix, 1 μL each of 1 pM/μL forward and reverse primers, 1 μL of 25 mM MgCl_2_, and 1 U of Taq polymerase. PCR was performed for an initial denaturation of 2 min at 95°C followed by 35 cycles of 45 s at 95°C, 45 s at the appropriate annealing temperature for each microsatellite primer pair as highlighted in Table 1 and 60 s at 72°C, and a final extension of 20 min. at 72°C.

### Gel Analysis

The developed SSR primers with 4% SFR (super fine resolution) agarose (AMRESCO, Solon, Ohio, USA) gel electrophoresis system running with 1× TBE buffer in 4°C using circulatory cool buffer system was used for better resolution of polymorphic differences between downy mildew isolates. The SSR amplification products were also analyzed using a LI-COR-NEN^® ^Model 4300 -dual-dye automated DNA Analyzer. Following amplification, an equal volume (5 μl) of PCR products of two sets of SSRs one labeled using the IRD-700 nm and another with IRD-800 nm fluorogenic forward primer were mixed. Basic fusion dye (2 μl) (LI-COR) was added to each pooled sample and the samples were denatured for 5 min at 95°C. Each sample (1 μl) was loaded on a 6.5% polyacrylamide gel containing 7 M urea. Gels were cast using LI-COR 25-cm plates with 0.25-mm-thick spacers and comb. Electrophoresis was performed at a constant power of 40 W and a constant temperature of 47.5°C for 2 h.

### Statistical analysis

Microsatellite fragments were scored as present (= 1) or absent (= 0) across the 34 *Peronosclerospora, Peronospora *and *Sclerospora *spp. isolates. The resulting binary matrix was used to construct a similarity matrix using the Jaccard coefficient, GS(*ij*) = *a*/(*a + b + c*) (17), where *a *is the number of fragments shared by *i *and *j*, *b *is the number of fragments present in *i *and absent in *j*, and *c *is the number of bands present in *j *and absent in *i*. Cluster analysis was performed using the unweighted pair group with arithmetic means method (UPGMA) [33]. PAUP 4.0* was used to generate 1000 bootstrap replicates for testing the reliability of the dataset and to draw a consensus tree [34]. Ordination analysis was performed to study the relatedness within a matrix by converting the pairwise distance into Eigen vectors and values. Cluster analyses, ordination analyses and the Mantel test were performed using NTSYSpc (NTSYS – for Numerical Taxonomy SYStems) version 2.1 [35].

## Results

A collection of 513 colonies, picked at random from primary transformation plates, was inoculated into 96-well culture plates for high throughput sequencing. Of the 513 clones sequenced, 170 (33%) were redundant with other clones in the same collection. A total of 73 out of 343 clones (21%) did not contain repeat motifs. Of the remaining 270 nonredundant clones, Sequence analysis showed that virtually all of the inserts contained microsatellite repeat motifs matching one or more of the biotinylated oligonucleotides used in the selection process. Out of 270 clones, 144 insert fragments (53%) were truncated within, or at the end of, a microsatellite repeat. Of the remaining 126 non redundant clones with unique sequences flanking both ends of the microsatellite repeats, 55 were selected for further analysis on the basis of the following criteria: (i) the length of the microsatellite was five or greater in dinucleotide and four in trinucleotide repeat units and; (ii) the unique 5' and 3' flanking sequences were both of suitable structure and composition for the design of efficient primers. Microsatellites with flanking sequences that were highly repetitive in nature and/or had high A+T rich nucleotides were eliminated.

Thirty six microsatellite loci containing a simple repeat type made up 66% of the DM collection, including 28 (55%) with dinucleotide repeat types and 6 (11%) with trinucleotide repeat types (three CAA and one each of GTT, AGG and ACA). Of the microsatellites containing a simple repeat type, 94% had "perfect" repeats (except DM9 and DM16), uninterrupted by nonrepeat nucleotides. The remaining 19 SSR loci (39%) of the collection were composed of "compound repeats" consisting of more than one repeat type at a single locus and all were interrupted by nonrepeat nucleotides and hence considered as "imperfect" repeats (Table 2). All the compound repeats were combinations of dinucleotide repeat motifs except DM45 (trinucleotide) and DM49 (combination of tetra and dinucleotide) repeat motifs. A total of 22 microsatellites with the CA/AC or GT/TG repeat type were the most abundant (40%) and the GA/AG or CT/TC type contributes 15% in our collection.

**Table 2:** Examples of different types of microsatelitte repeat structures identified in *Peronosclerospora sorghi *– pathotype. 3

Microsatelitte loci	Type	Repeat structures
DM2	simple and perfect repeat	GT GT GT GT GT GT GT GT

DM16	simple and Imperfect repeat	TG TG TG TG TG TG TG TG TG T**A**TG TG TG TG TG TG TG TG TG TG TG TG TG TG TG TG TG TG TG TG TG TG

DM13	compound and imperfect	CACACACACACACACA**TCTG**TATATATA

Bold and underlined are interrupted non repeat nucleotides makes the SSR loci imperfect

Of the 55 cloned SSR sequences queried in BLASTN searches of the NCBI non-redundant genomic and EST databases(as of July 4, 2008), only four clones DM21, DM28, DM53 and DM55 were found to show similarity to previous entries and these were to sequences of *Phytophthora *and *Pythium *species which are also plant pathogenic oomycetes. Details are presented in table 3. These results indicate that two of the cloned sequences (DM53 and DM54) isolated from *P. sorghi *contain incomplete portions of genes found in known retrotransposon elements.

**Table 3:** Results of BLAST Query – SDM fungal microsatellites showing homology against the NCBI database

SSR loci	Locus, Species, & Most related sequence (Genomic DNA/EST)	Query coverage (%)	Identity (%)	E-value*
DM21	DQ645744, *Phytophthora ramorum *(Sudden oak death agent – Fungi), transposon GypsyPr-2 reverse transcriptase gene – **Genomic DNA**	26	96	3e–20

DM28	AF312890, *Phytophthora cinnamomi *SSR sequence – **Genomic DNA**	45	90	4e–36

DM53	DQ645744.1 – *Phytopthora ramorum *transposon GypsyPr-2 reverse transcriptase gene	96	78	4e–70
	AY830104, (GypsyPi-3a), AY830105 (GypsyPi-3b) *Phytophthora infestans *– retrotransposon	93	96	3e–14
	AF490339, *Phytophthora infestans *– Gypsy like retrotransposon,	87	90	2e–09
	DQ645742, *Phytophthora sojae *transposon GypsyPs-1A reverse transcriptase gene	78	90	4e–07

DM55	AY564217, *Phytophthora ipomoeae*	54	90	6e–32
	EU427470.1 – *P. ramorum*	52	89	3e–30
	AY564219 – *P. andina*, AY564218-*P. phaseoli*, AY564213 to AY564216 – *P. mirabilis*, AY564209 to AY003911 – *P. infestans *&DQ832717 and AY564221- *Phytophthora sojae*	54	89	3e–30
	- **mitochondrion DNA**			
	
	ES287433 and ES286374*Phytophthora brassicae*	54 54	88 88	2e–30 2e–29
	CV935202*Phytophthora infestans *(potato late blight)	54	86	1e–26
	EL774547, EL777768, EL775494, EL779446, EL781147 and EL775427*Pythium ultimum*			
	- **ESTs**,			

*E value or the Expect value is a parameter that describes the number of hits one can "expect" to see just by chance when searching a database of a particular size

Primers were designed for the 55 selected microsatellite loci using the criteria described in materials and methods. These primers (for Table 4 see additional file 1) had an average length of 20.7 nucleotides, with an average G+C content of 49% and annealing temperature in a range of 50–55°C. Predicted cloned products ranged in size from 106 (DM52) to 249 (DM21) bp. These 55 new microsatellite loci described in Table 4 (see additional file 1) were designated as "DM" (Downy Mildew). One of the 55, DM15 was not amplified in any of the pathotypes when resolved either in SFR agarose or LI-COR polyacrylamide gels. DM17, 27, 40, 41, 45 and 48 were resolved with faint expression in SFR agarose gels and with fine resolution in LI-COR polyacrylamide gels. When used with 34 *Peronosclerospora *and *Sclerospora *spp isolates, a total of 260 alleles were obtained using 54 microsatellites primer combinations in LI-COR polyacrylamide gels, with an average of 4.8 polymorphic markers per SSR. In comparison, only 128 alleles were visible using 48 SSRs in SFR agarose gels with an average of 2.7 polymorphic markers per SSR across the same set of isolates. Four SSRs (DM12, 19, 30 and DM38) showed monomorphic expression in SFR agarose gels but polymorphisms were detected in LI-COR poly-acrylamide gels. SFR agarose gel data for 54 SSRs across species were not used further in this study.

**Figure 1:**
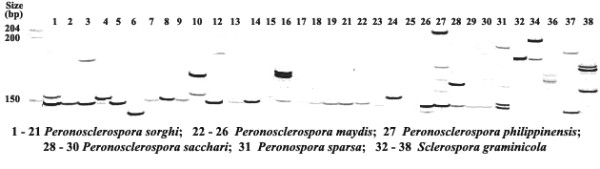
**Licor gel image of 34 isolates of different downy mildew species using DM 9 SSR primers**. Simple sequence repeat polymorphism fingerprint of 34 isolates of different species of *Peronosclerospora, Peronospora *and *Sclerospora *after amplification with SSR primers of DM 9. Lanes 1 through 38 represent the downy mildew isolates of different species encoded in table 1. Molecular weights are indicated in base pairs to the left.

Of the 54 SSR primer pairs, 50 produced amplicons in *Peronosclerospora sorghi*, 41 in *P. maydis*, 29 in *P. sacchari*, 33 in *P. philippinensis*, 30 in *Peronosopora sparsa *and 37 in *Sclerospora graminicola *downy mildew isolates (Table 5). Twelve SSR primer pairs (DM 9, 12, 13, 14, 16, 19, 30, 31, 35, 49, 53 and DM54) amplified products in all six species. A total of 15 (DM 3, 8, 11, 13, 18, 27, 31, 39, 40, 45, 50, 52, 53, 54, & DM 55) and seven (DM13, 23, 24, 45, 48, 50 and DM53) produced unique bands in *P. philippinensis *and *P. sparsa *respectively. Representative examples of the distinct banding differences for amplification products obtained with 34 *Peronosclerospora, Peronospora *and *Sclerospora *spp. isolates using the DM9 microsatellite primer pair are shown in Fig. 1.

**Table 5:** Amplification details of all 54 SSR loci in different downy mildew species

*Peronosclerospora. Sorghi*	*P. maydis*	*P. sacchari*	*P. philippinesis*	*Peronospora sparsa*	*Sclerospora graminicola*
DM 1, 2, 3, 4, 5, 6, 7, 8, **9**, 10, 11, **12**, **13, 14**, **16**, 17, 18, **19**, 20, 21, 22, 23, 24, 25, 26, 27, 28, **30**, **31**, 32, 33, 34, **35**, 36, 38, 40, 42, 43, 44, 45, 46, 47, 48, **49**, 50, 51, 52, **53**, **54**, & DM 55	DM 1, 5, 6, 7, 8, **9**, 11, **12**, **13, 14**, **16**, **19**, 20, 21, 23, 24, 26, 27, 28, **30**, **31**, 32, 33, 34, **35**, 36, 37, 38, 40, 41, 43, 44, 45, 46, 47, **49**, 51, 52, **53**, **54**, & DM 55	DM 5, 7, 8, **9**, **12**, **13, 14**, **16**, 17, **19**, 23, 26, 28, 29, **30**, **31**, 33, 34, **35**, 37, 38, 43, 47, **49**, 51, 52, **53**, 54, & DM 55	DM 3*, 5, 7, 8*, **9**, 10, 11*, **12**, 13*, **14**, **16**, 18*, **19**, 22, 26, 27*, 28, **30**, 31*, 33, **35**, 39*, 40*, 44, 45*, 46, 47, **49**, 50*, 52*, 53*, 54*, & DM 55*	DM 2, 3, 7, **9**, **12**, 13*, **14**, **16**, 17, **19**, 20, 22, 23*, 24*, 25, 27, **30**, **31**, **35**, 36, 40, 41, 43, 45*, 48*, **49**, 50*, 51, 53*, & DM **54**	DM 2, 3, 4, 8, **9**, 10, 11, **12**, **13, 14**, **16**, 17, 18, **19**, 20, 22, 23, 25, 27, 29, **30**, **31**, **35**, 37, 38, 39, 40, 42, 45, 46, 48, **49**, 51, 52, **53**, **54**, & DM 55

Total: 50	41	29	33	30	37

Primers in bold letters are cross- amplified in all species

* Primer combinations expressed unique bands differences from other species

**Figure 2:**
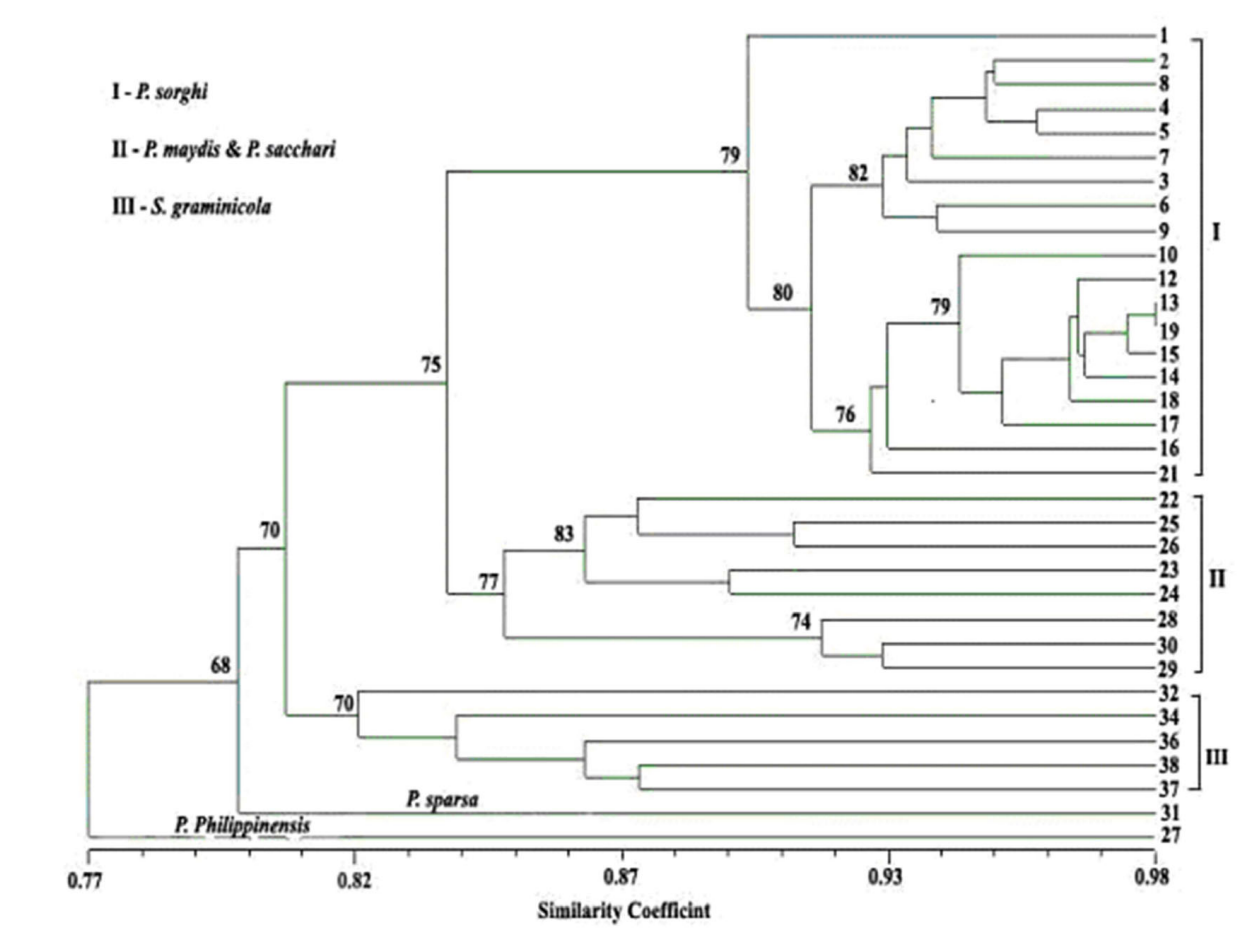
**Cluster analysis**. Dendrogram showing the clusters of 34 *Peronosclerospora, Peronospora *and *Sclerospora *isolates using 54 sorghum downy mildew microsatellites. Percentages from 1000 bootstrap replications are shown near the branches of each cluster.

**Figure 3:**
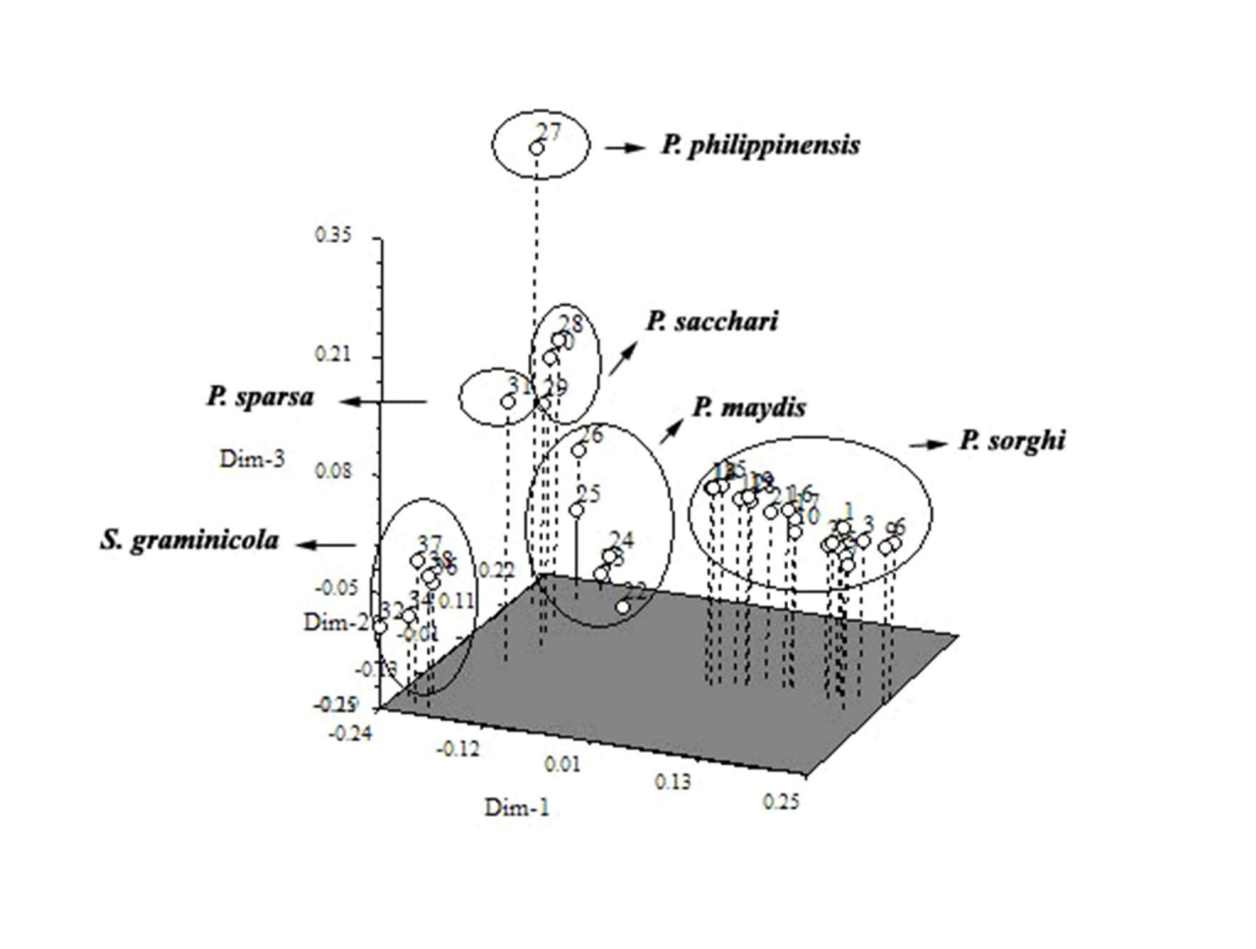
**Principal coordinate analysis (PCA)**. Three-dimensional display of 34 *Peronosclerospora, Peronospora *and *Sclerospora *isolates based on the combination of data obtained with 54 SSRs. Dimensions 1, 2 and 3 are accounted 86% of the variation observed.

A presence (1) or absence (0) binary matrix containing 260 clear amplicons from 54 SSRs was used to generate the genetic similarity estimates. The similarity co-efficient ranged from 0.77 to 0.98 indicating narrow genetic distance among the isolates of the different species studied. The dendrogram constructed using UPGMA (Fig. 2) summarizes the interrelationship observed among 34 isolates of different downy mildew species. With the similarity coefficient greater than 0.84, all the 34 isolates were grouped into three distinct clusters. In the cluster I, 18 *Peronosclerospora sorghi *isolates including pathotype P3 (metalaxyl sensitive) and other metalaxyl resistant isolates of P3 and P6 pathotypes were all grouped together with a confidence limit of 80%. Within cluster I, Metalaxyl sensitive isolates of P1 pathotype (#1, 16 and 21 from Fig. 2) were distinctly separated from others with a confidence limit of 79% and 76%. Five isolates of *P. maydis *and three isolates of *P. sacchari *were grouped in two sub clusters with confidence limits of 83 and 74% respectively in cluster II and five isolates of *Sclerospora graminicola *were grouped in cluster III with confidence limit of 70%. The remaining *P. sparsa *and *P. philippinensis *isolates were not grouped into any of these three clusters and showed their distinct uniqueness from other species. Principal coordinate analysis (PCA) was also performed to show the relationship among the 34 isolates as a three dimensional display. In this analysis the first two principal components (having eigen value >1) explained 86% of the total variation. Like the UPGMA clustering dendrogram, PCA analysis placed isolates of different species of downy mildew into distinct positions (Fig. 3).

## Discussion

The *Peronosclerospora *spp. that cause downy mildew of sorghum and maize and other gramineous hosts include some of the most destructive pathogens in the tropics and subtropics [36] and some of them infect more than one important crop. Of the 10 recognized species of fungi that cause downy mildew diseases of maize, 5 currently are in the genus *Peronosclerospora *[37,38]. The confusion over nomenclature of specific pathogens involved in these diseases is a major problem in global evaluation of the downy mildews caused by *Peronosclerospora*. It is difficult to determine if research results and disease control methods developed in one area of the world might be applicable to another. In several instances, taxonomy within the genus is confused because, at most, only slight differences in morphology of recognized species exist [39]. With the result of traditional approaches [40] combined with utilizing molecular techniques including isozyme analysis [41] and PCR (Polymerase Chain Reaction, [14]) the maize strain of *P. sorghi *from Thailand was given specific rank as *P. zeae *[13]. Legg [42] has outlined the advantages of some of the molecular methods for studying the *Peronosclerospora *spp. A maize strain of *P. sorghi *was reported in southern Nigeria [43]. However, Yao et al. [14] using PCR, were unable to differentiate an isolate of SDM from maize in southern Nigeria from sorghum/maize-infecting strains and the identity of this pathogen remains unknown. The problems described warrant development of simple and powerful molecular diagnostic tools for constant monitoring of this disease. Since microsatellite regions are highly mutable due to their propensity of addition and deletion of repeat copies they can be used to differentiate within and between related taxa, even at the level of individual isolates in a single species. Simple DNA- profiling methods based on microsatellite variability provide possibilities to identify individual genotypes for studies in population genetics, ecology and taxonomy [44].

### Characterization of microsatellite loci

In the present study, a total of 55 microsatellite loci were isolated from *Peronosclerospora sorghi *– pathotype P3 DNA enriched for simple sequence motifs. We observed 33% internal redundant motifs, likely consequences of using two PCR amplification steps during the isolation process. Of the sequenced clones with an insert, over 79% contained microsatellites. This is a significant enrichment as compared to traditional microsatellite isolation protocol [45]. However, the relatively small fragment size average resulting from the use of three restriction enzymes in the initial fragmentation (*Hae*III, (GGCC) targets, *Rsa*I, (GTAC) and *Dra*I (AAATTT)) may explain why 144 insert fragments out of 270 clones (53%) were truncated within, or at the end of, a microsatellite repeat. This result is in accordance with the CM collection [46], in which genomic DNAs were mechanically fragmented using a high-pressure nebulizer, and a substantial portion of the clones were truncated within the microsatellite. The most frequent dinucleotide microsatellite motifs detected in our libraries were (CA/AC)_n _or (GT/TG)_n_. followed by (GA/AG)_n _or (CT/TC)_n_. In earlier studies, (CA)_n _repeat motifs [47] and (AG)_n _repeat motifs [48] were also detected from the ascomycetes, *Podospora anserina *and *Lobaria pulmonaria *respectively. Approximately 39% of all isolated loci were composed of complex intermixed motifs, which were rarely arranged in a tandem way. This pattern, known as cryptic simplicity [49], is common in larger eukaryotic genomes [50]. Microsatellites identified with trinucleotide repeats (CAA, AGG and ACA) were found in this study with low frequencies, but seem to differ from those typically found in 14 fungal genomes (AAG, AAC, AGC, and ATC) [51]. One trinucleotide repeat clone (DM45) included multiple repeats in a complex pattern but no significant homology was found to other sequences currently in GenBank.

Determining if microsatellites will be polymorphic can only be assessed empirically, however, two considerations can help predict if polymorphism is likely. First, as slippage during replication increases with the number of tandem repeats [52], loci in which the motif is iterated at least eight times are desirable. Second, it may be possible to predict the stability of a given microsatellite motif by assessing the likelihood of "expandability" [53]. Microsatellites reflect a balance between expansion and contraction, but while there is evidence of a bias toward increasing microsatellite size [54], very long tandem repetitions are rare [55]. In contrast, in our study, (CA/AC)n and (GT/TG)n were commonly repeated more than 20 times, suggesting expansion to these lengths is not limited. The presence of these motifs in most fungal and oomycete genomes suggests they would be useful microsatellites to target in different species where genome data are not available.

### Retrotransposable Elements

The presence of retrotransposon-like elements (mobile genetic elements) plays an important role in genome evolution and such elements constitute about 5 to 10% of the genome in eukaryotes [56]. DM55 (ACA)_19 _showed significant sequence homology to mitochondrial sequences in several *Phytophora *species (Table 3). Two of the *P. sorghi *SSR sequences were found to have a degree of similarity to reverse transcriptases, suggesting an origin from retrotransposons possibly present in a progenitor species. In this study, *P. sacchari *and *S. graminicola *isolates were not amplified with DM 21, whereas, DM53 cross amplified in all the species studied. These results suggest the GypsyPr-2 reverse transcriptase-like gene sequence as defined in *Phytopthora ramorum *was not present in *P. sacchari *or *S. graminicola *isolates. However, sequences similar to the *Phytopthora infestans *GypsyPi-3a and GypsyPi-3b retrotransposon-like and to the GypsyPs-1A sequence from *Phytopthora sojae *were present in all *Peronosclerospora, Peronospora *and *Sclerospora *downy mildew isolates. Primers and probes built to take advantage of the presence of unique differences in such retrotransposon sequences can be useful for strain/race identification. Examples include the work by Sastryet al. [57] with *S. graminicola *and Becker et al. [58] in the basidiomycete *Chondrostereum purpureum*.

### Gel analysis

Resolving microsatellite amplicons in polyacrylamide gels using a LI-COR high throughput sequencing system with fluorogenic primers results in significantly more useful bands than resolving the same products in 4% SFR gels stained with ethidium bromide. For example DM12, 19, 30 and 38 gave monomorphic expression in SFR agarose gels but showed alleles with polymorphic differences in LI-COR poly-acrylamide gels. Taken together, from combined data across 34 *Peronosclerospora, Peronospora *and *Sclerospora *spp. isolates, an average of 4.8 polymorphic markers were detected per SSR in LI-COR poly-acrylamide gels versus 2.7 in SFR agarose gels. Thus the use of LI-COR poly-acrylamide gels with fluorogenic labeled primers was more efficient in separating bands which reflect clear cut polymorphisms. Fingerprinting using 54 SSR primer pairs over all the isolates resulted in 260 diagnostic alleles. Although it is assumed that the majority of size variants (100 to 350 bp) was due to variation in the number of the repeat motifs, this has not been verified by sequencing. Insertions, deletions and base substitutions in the flanking regions [59] might also account for variation in fragment length, especially when amplicons from different species are compared.

### Cross-species amplification

It has been shown that microsatellite primers developed for a distinct species can be useful for genetic analysis in closely related species [60], but successful transferability depends upon the evolutionary distance between source and target species [61]. Dutech et al. [62] reported relatively low cross-species transferability of microsatellites in fungi. Within genera, only 34% of the loci tested could be transferred, which appears much lower than in animals or plants. Here, twelve SSRs (DM 9, 12, 13, 14, 16, 19, 30, 31, 35, 49, 53 and DM54) were cross amplified in samples from 6 species of downy mildew-causing oomycetes. Successful cross-amplification simply indicates that the flanking regions are conserved across the species studied, but it does not tell anything about the character and structure of the fragment and additional sequencing will be necessary to clarify the structure of these fragments. Similar findings with successful cross amplification of SSRs in closely related species were reported for lichen-associated fungi [48] and in for a variety of fungal species [51]. Another interesting feature evident from our study is the distinctness of different species as revealed by unique banding patterns with different SSRs as evinced clearly from Fig. 1. Unique bands were noted with DM 3, 8, 11, 13, 18, 27, 31, 39, 40, 45, 50, 52, 53, 54, & DM 55 for *P. philippinensis *and DM13, 23, 24, 45, 48, 50 and DM53 for *P. sparsa*. While these amplicons may be useful for diagnostics based on unique banding patterns, the information for *P. philippinensis *and *P. sparsa *is based on single isolates, so data from more independent, species-verified samples will be required to assess the degree of variation within natural populations. Based on the species-specific clustering of isolates where multiple samples were available, distinctive fingerprints can be anticipated.

### Cluster analysis

In the present study, SSR markers revealed using high throughput LI-COR-NEN^® ^Model 4300 -dual-dye automated DNA Analyzer have been used to resolve cryptic genetic variation of closely related different downy mildew species that have been impossible to resolve with morphological systematic characters. SSR fingerprinting was conducted using 54 primer pairs and genomic similarity analyses derived from qualitative data grouped isolates of samples of downy mildew according to host specificity (sorghum, maize, sugarcane, pearl millet and rose) whose taxa had been uncertain based on morphological criteria. In this study, metalaxyl sensitive P3 isolate (#2 in Fig. 2) in cluster I grouped with all other metalaxyl resistant isolates, suggested that metalaxyl resistance may have originated in a P3 isolate to give rise to the recent outbreak, consistent with results of based on AFLP molecular characterization [15].

However, mating and recombination involving metalaxyl-resistant genotypes and other isolates, along with selection in favor of resistance would narrow the genetic base and can account for the high similarity (approximately 89%) among all 19 *P. sorghi *isolates in cluster I. *P. maydis *and *P. sacchari *isolates in cluster II were grouped into two sub clusters with high similarity coefficient (approx. 82%) indicates that corn and sugarcane downy mildew isolates have narrow genetic differences, showing more genetic similarities with each other than other *Peronosclerospora *species. The single isolates of *P. philippinensis *and *P. sparsa *were not grouped with any of the three clusters and showed their uniqueness due to their distinct banding pattern with different microsatellite primer combinations as detailed earlier. The three-dimensional comparison is compatible with cluster analysis and provides a good visual comparison of the genetic similarities and differences of the isolates. Fig. 3 clearly depicted the unique identity of *P. philippinensis *from other species. This display shows that the genetic distance between *P. philippinensis *and *P. sacchai *is very narrow. Similar findings with close phylogenetic relationship was recorded through isozyme analysis between *P. philippinensis *and *P. sacchari *isolates from Taiwan [26,63] and by RFLP patterns by [13]. Further study is clearly needed to confirm this relationship. Unfortunately, the select agent status of *P. philipinnensis *has made the obtaining of additional samples, even of DNA, impossible to date.

## Conclusion

Microsatellite primer sets developed from *P. sorghi *sequences proved to be useful for all downy mildew species analyzed and are likely to be increasingly developed and applied to studies of pathogen epidemiology, population biology, and genomics. The diversity of microsatellite motifs gave each species a unique "signature" of repeat distributions. Subsets of the 54 newly developed SSRs may be very useful for rapid and efficient identification and genetic analysis of the natural populations and host range of these obligate oomycetes. The unique banding pattern of *P. philippinensis *from fifteen (DM 3, 8, 11, 13, 18, 27, 31, 39, 40, 45, 50, 52, 53, 54, & DM 55) and *P. sparsa *from seven (DM13, 23, 24, 45, 48, 50 and DM53) SSRs make these primers useful as diagnostic markers for the respective species. In future studies it will be possible to focus on the population diversity and recombination within and between native and introduced populations of the closely related species. These distinct fingerprinting profiles can be used as diagnostic tools to formulate breeding strategies targeting host resistance to local pathogen populations and for monitoring the emergence of new virulent races.

## Authors' contributions

RP performed the capturing of microsatellites, data analysis and wrote the manuscript. PN and UKR provided lab facilities and assisted in capturing microsatellites. RP and SRE carried out all gel analyses. EGN performed sequence analysis. LKP, GNO, DGL and CWM provided the source materials. DGL and CWM directed and oriented the project and revised the manuscript. All authors read and approved the final manuscript.

## Supplementary Material

Additional file 1 **Information on 55 SDM (*Peronosclerospora sorgi*) microsatellites loci.** Microsatellite loci repeat motif, primer sequence (forward and reverse), allele size, annealing temperature (T_a_), number of alleles detected and gene bank accession numbers. Click here for file
